# Effect of Dietary Supplementation of Immunobiotic *Lactiplantibacillus* *plantarum* N14 Fermented Rakkyo (*Allium chinense*) Pickled Juice on the Immunocompetence and Production Performance of Pigs

**DOI:** 10.3390/ani11030752

**Published:** 2021-03-09

**Authors:** Md. Aminul Islam, Kenji Hashiguchi, A.K.M. Humayun Kober, Kyoko Morie, Binghui Zhou, Mikado Tomokiyo, Tomoyuki Shimazu, Hisashi Aso, Julio Villena, Yoshihito Suda, Haruki Kitazawa

**Affiliations:** 1 Food and Feed Immunology Group, Laboratory of Animal Products Chemistry, Graduate School of Agricultural Science, Tohoku University, Sendai 980-8572, Japan; aminul.vmed@bau.edu.bd (M.A.I.); humayuna2002@yahoo.com (A.K.M.H.K.); k-morie@oenon.jp (K.M.); iiiiisabelllll@gmail.com (B.Z.); mikado0403@gmail.com (M.T.); 2 Livestock Immunology Unit, International Education and Research Center for Food and Agricultural Immunology (CFAI), Graduate School of Agricultural Science, Tohoku University, Sendai 980-8572, Japan; asosan@tohoku.ac.jp; 3 Department of Medicine, Faculty of Veterinary Science, Bangladesh Agricultural University, Mymensingh 2202, Bangladesh; 4 Research and Development Group, Momoya Co., Ltd., Saitama 344-8522, Japan; hashiguchi@momoya.co.jp; 5 Department of Dairy and Poultry Science, Faculty of Veterinary Medicine, Chittagong Veterinary and Animal Sciences University, Chittagong 4225, Bangladesh; 6 Department of Food Science and Business, School of Food Industrial Sciences, Miyagi University, Sendai 982-0215, Japan; shimadut@myu.ac.jp; 7 Laboratory of Animal Health Science, Graduate School of Agricultural Science, Tohoku University, Sendai 980-8572, Japan; 8 Laboratory of Immunobiotechnology, Reference Centre for Lactobacilli, (CERELA-CONICET), Tucuman 4000, Argentina; 9 Department of Food Resource Development, School of Food Industrial Sciences, Miyagi University, Sendai 982-0215, Japan

**Keywords:** pickled juice, immunobiotics, gut health, Immune function, meat production, *Lactiplantibacillus plantarum* N14

## Abstract

**Simple Summary:**

Fermented pickle of Rakkyo (Japanese leek) is a popular dish because it has several proven human health benefits. We tested the effects of dietary adding *Lactiplantibacillus plantarum* N14 fermented rakkyo pickled juice on the immunocompetence and production performance of pigs. The results showed that the use of 5% mixture of fermented rakkyo pickled juice and the rakkyo residues in the porcine feed could be a cost-effective approach to promote the immune-health and production performance of pigs. This approach might contribute to exploring the sustainable management of food waste through utilizing them as livestock feed supplement.

**Abstract:**

Rakkyo (*Allium chinense*), is a Japanese leek that is primarily used to make a popular sweet or sour pickled dish. Lactic acid bacteria are often involved in the preparation steps of fermented pickles, which helps in the effective preservation of the natural bioactive compounds of fruits and vegetable, and thereby exert several health benefits including immunomodulation and growth performance. This work aimed to evaluate the in vivo effects of adding *Lactiplantibacillus plantarum* N14 fermented rakkyo pickled juice as feed supplement on the immunocompetence and production performance of pigs. We first analyzed the nutritional composition, which revealed that the proportion of protein, lipid, and water-soluble fiber content were estimated as of 4%, 5%, and 5% in rakkyo residual liquid or juice, while 22%, 15% and 14%, respectively, were estimated in rakkyo residual powder. For the in vivo feeding trials, three groups of pigs were treated either with 5%, 20%, or 40% mixture (*v/v*) of fermented rakkyo pickled juice and the grinded residual liquid supplemented in the drinking water in addition to standard feed. The results of the feeding trials showed that the administration of a juice mixture of 5% or 20% (fermented pickled juice and residual liquid) had a similar trend of effects in improving the complement activity, phagocytic activity and leucocytes counts in the peripheral blood when compared to pigs fed with 40% mixture or untreated controls. Those changes were related to an improved resistance to enteric infections. Moreover, animals receiving a mixture of fermented pickled juice and fermented rakkyo residues had a higher growth rate and carcass quality than controls. The results suggested that the use of 5% mixture of fermented rakkyo pickled juice and the residual liquid through drinking water could be a cost-effective approach to promote the immune-health and production performance of pigs. This approach would contribute not only to the sustainable management of food wastes but also to the application of a value-added feed supplement for the promotion of animal health and production.

## 1. Introduction

Rakkyo (*Allium chinense*) is a root vegetable having a crisp texture with a subtly sweet and sour taste quite similar to shallot. The rakkyo bulb looks like a small piece of garlic, but this Japanese leek belongs to the Amaryllidaceae family. Rakkyo are sweet, pickled scallions that are served alongside Japanese curry. Among the different pickled products on the market, fermented rakkyo pickle is becoming increasingly accepted by consumers, particularly in Japan [1]. Due to the high human consumption of pickled dish, the spent liquor or juice of rakkyo pickles are producing the bulk of food waste.

Pickling is an ancient preservation technique for fruits and vegetables by either anerobic fermentation or immersion in vinegar [2,3]. The pickling process helps in the effective preservation and restoration of natural bioactive compounds of fruits and vegetables for an extended period of time. Fermentation is a slow decomposition process of organic substances induced by microorganisms or enzymes that essentially convert carbohydrates to alcohol or organic acids [2]. Lactic acid bacteria (LAB) are often involved in the preparation steps of fermented pickles, which ferment sugars in the food and produce lactic acid, and thereby prevent the growth of food poisonous bacteria and molds. Like other vegetables such as cucumber, cabbage, olive and onion, rakkyo pickle is fermented by LAB including *Lactiplantibacillus plantarum* which can grow in a low concentration of salts [4,5,6]. The availability of certain specific nutrients such as vitamins and minerals, and the acidic nature of fruits and vegetables provides conducible medium for the growth of LAB. Moreover, the biochemical composition of rakkyo pickled juice may be affected by different processing steps and storage methods, and it is crucial for exerting beneficial effects to the host [4,7,8]. Therefore, the analysis of nutritional composition, in particular, the detailed profiling of sugar and amino acid contents of fermented rakkyo pickled juice and fermented rakkyo residual is of interest.

Several members of *Lactobacillus* spp. have been used as food-grade microorganisms because of their long and documented history of safe use in fermented foods [9,10]. During the pickling of rakkyo, growing LAB secrete exopolysaccharide (EPS) and other metabolites in the juice used for fermentation [11,12]; as such, fermented pickled juice becomes enriched with bioactive compounds having immunomodulatory potential [9]. Strains of *L. plantarum* have shown to be efficient in food fermentation and to exert beneficial effects to host health in a strain-dependent manner. Therefore, every lactobacilli strain must be evaluated individually in terms of their potential use in the development of functional fermented foods. An increased awareness about healthy food has led to an increasing interest in natural foodstuff and nutraceuticals such as using probiotic supplements in livestock feed [13]. The dietary application of probiotics with a liquid application system has recently been reported have a significantly positive influence on the meat quality and physicochemical characteristics of pork [14]. Probiotic lactobacilli have long been used for improving growth performance, feed conversion efficiency, carcass weight and pork quality, intestinal microbiota, gut health, and immune system of pigs [15,16,17]. We previously demonstrated that immunobiotic *L. jensenii* TL2937 is able to promote the immune-health, growth performance, and productivity in post-weaning pigs [18]. On the other hand, it was reported that a daily oral intake of *L. plantarum* N14 reduced the illness of seasonal allergic disease in humans [19,20]. The pickled juice used for this study was derived from rakkyo pickles which were fermented by using the starter culture of *L. plantarum* N14 [8]. Studies have shown that the N14 strain is able to secrete both acidic and neutral EPS, which exert the anti-allergic and immunostimulatory abilities [19,21]. In a recent in vitro study, we also demonstrated that *L. plantarum* N14 and its acidic EPS can reduce inflammation through the modulation of the toll-like receptor (TLR) signaling in porcine intestinal epithelial (PIE) cells [8]. Therefore, whether *L. plantarum* N14 present in the spent pickled juice has similar beneficial health effects in pigs is of great interest.

Besides the effective management of the post-consumption food waste of fermented rakkyo pickles through sustainable utilizing as feed supplements, pickled juice may serve as a good carrier of immunobiotic *L. plantarum* N14 and its secreted metabolites having antioxidant capacity [1,8]. On the other hand, the industrial processing of rakkyo vegetables for the mass-scale pickle production generates high volumes of residual by-products (peels, stalk, leaves, etc.), which can also be used as animal feedstock. The rakkyo residues contain a diverse group of prebiotic compounds which attract and stimulate the growth of immunobiotics carried by pickled juice when added into the feed, and thereby develop a synergistic combination of prebiotics and immunobiotics called ‘immunosynbiotics’ [22]. We therefore hypothesized that the spent pickled juice could be used as value-added feed supplement for the healthy livestock growth. Understanding the relationship between feed, beneficial microorganism, and health of the animal is important to improve the quality of feed, increased productivity, and prevention of several diseases. Therefore, the objective of the present study was to evaluate the beneficial effects of feeding of *L. plantarum* N14 fermented rakkyo pickled juice to immunocompetence and production performance of pigs.

## 2. Materials and Methods

### 2.1. Preparation of Rakkyo Pickled Juice by Lactic Acid Fermentation

Rakkyo (*Allium chinense*) was processed to prepare the pickles through fermentation by using the immunobiotic *Lactiplantibacillus plantarum* No.14 as starter culture (LP14 starter fermentation product/2009, Momoya Co. Ltd., Saitama, Japan). The fermented rakkyo pickles were subjected to colander separation, and the liquid portion was then considered as fermented rakkyo pickled juice and the solid part as residues. The rakkyo residues were subsequently subjected to grinding followed by centrifugation to separate into residual solid and rakkyo residual liquid. Finally, a mixture (*v*/*v*) of an equal volume of fermented rakkyo pickled juice and rakkyo residual liquid was used for the in vivo feeding trial. The concentration of *L. plantarum* No.14 in the fermented rakkyo pickled juice was 1.0 × 10^7^ CFU/mL.

### 2.2. Evaluation of Nutritional Composition of Fermented Rakkyo Residues

Samples were taken from the solution of fermented rakkyo residual powder, and fermented rakkyo residual liquid without adding food-grade dextrin, followed by a comparative analysis of nutritional ingredients. The proportion of total protein, lipid, ash, moisture and water soluble- and insoluble-fiber, and total sugar were estimated. In addition, individual sugar composition and amino acids content were analyzed.

The moisture and solid fiber percentage of rakkyo residual liquid was determined by the normal pressure heating and drying method. The percentage of water soluble and insoluble dietary fiber content was also determined by Proski modified method [23]. The total lipid content was estimated by the procedure established by Folch et al. [24]. The ash content was determined gravimetrically by incinerating 1 ± 0.1 g of oven-dried powder sample in Muffle furnace at about 550 °C for 6 h and the results were expressed in percentage [25]. The protein content of raw and fermented rakkyo was determined by the standard procedure [26]. Finally, the total sugar was estimated by the formula [100 − (protein + lipid+ ash + water + water-soluble dietary fiber + insoluble dietary fiber)] as described by Menezes et al. [27].

### 2.3. Quantitative Analysis of Polysaccharide Composition

The 80% ethanol (*v*/*v*) was added to the sample, and the obtained insoluble matter was dehydrated with acetone and dried. Then, 10 mL of 72% sulfuric acid (*w*/*w*) was added, and the mixture was left to stand at 20 °C for 4 h, diluted with 140 mL of water, and hydrolyzed in a boiling water bath for 2 h. After cooling to room temperature and neutralizing with a 30% sodium hydroxide solution (*w*/*v*), the volume was adjusted to 250 mL with water and filtered. This filtrate was passed through Sep-Pak plus Accell QMA and Sep-Pak plus Accell C18 (Nippon Waters, Tokyo, Japan), and the filtrate was filtered through a membrane filter with a pore size of 0.45 μm was used as a test solution. Different types of neutral polysaccharides were determined by High Performance Liquid Chromatography (HPLC).

For estimating the ranges of molecular weight of identified sugars, molecular weight standards were dissolved in a 0.1 mol/L sodium nitrate solution. A sample of 0.01 g was collected, and 10 mL of a 0.1 mol/L sodium nitrate solution was added. After standing at room temperature overnight, it was heated in a boiling water bath for 10 min. After allowing to cool, the solution was filtered through a membrane filter having a pore size of 0.45 μm. The obtained solution was used as a test solution, and the solution was measured by HPLC using a size exclusion column. The obtained results were analyzed using a 480-degree data station GPC program (System Instruments, Tokyo, Japan).

### 2.4. Amino Acid Analyses

The amino acid content of the fermented rakkyo pickled juice and residual powder was determined by high-performance liquid chromatography (HPLC, Merk-Hitachi L-7400) following the method previously described [28]. For this, 150 mg of samples were subjected to acid hydrolysis with 5 mL of 6 M HCl under nitrogen atmosphere for 24 h at 110 °C. Each hydrolysate was washed into a 50 mL volumetric flask and made up to the mark with Milli-Q water. The dried mass was washed with Milli-Q water and residue. The amino acids were subjected to HPLC (Merk-Hitachi L-7400) analysis after derivatization with 6-aminoquinolyl-N-hydroxysuccinimidyl carbamate (AQC). AQC was purchased as part of an AccQFluor Reagent kit from Waters. For derivatization, 10 μL of sample or amino acid standard mixture combined with 70 μL of 200 mM borate buffer (Water AccQFluor Borate Buffer, Waters, Milford, MA, USA). The samples were mixed, and 20 μL of AccQFluor reagent (AQC dissolved in acetonitrile) was added. After an immediate mixing, the samples were heated for 10 min at 55 °C to degrade a tyrosine byproduct. For analysis, samples of 5 μL were injected into the HPLC system. This consisted of a Waters 2690 Separations Module (Waters Assoc., Milford, MA, USA), a Jasco FP-920 fluorescence detector (Jasco Corp., Tokyo, Japan) (an excitation wavelength at 250 nm, an emission wavelength at 395 nm, and a flow cell of 5 μL), and a computer with Waters Millennium 32 Chromatography Manager, version 3.00. The separation of the AQC derivatives was carried out using a 3.9 × 150 mm AccQTag column (Waters, WAT052885, Milford, MA, USA), held at 37 °C and gradient elution. Three eluents were used. Eluent A consisted of 140 mM sodium acetate with 17 mM triethylamine, titrated to pH 5.05 with phosphoric acid. A total of 1 mg of disodium EDTA per ml and 0.001% sodium azide were added. Eluents B and C were acetonitrile and Milli-Q water, respectively. The flow rate was 1.0 mL/min.

### 2.5. Estimation of Microbial Loads in the Fermented Rakkyo Pickled Juice

The determination of microbial load was carried out by following the terminal restriction fragment length polymorphism (T-RFLP) method described by Caffaro-Filho et al. [29].

### 2.6. Selection and Management of Study Animals

For the in vivo feeding trial, a total of 20 healthy crossbred (Landrace × Yorkshire × Duroc) piglets were selected, allotted into four groups, and then housed in four separate sheds. The piglets weighed at 7 ± 0.29 kg at the start of experiment. To exclude the family effect, piglets were taken from litters of four sows including maximum one piglet of either sex from each sow. Male piglets included in this study were castrated at the first few weeks of age with adequate local anesthesia following the code of animal welfare ethics. After weaning, all piglets were raised and fattened with the administration of standard diet ad libitum without antimicrobial supplement. All pigs were maintained for this study from three weeks of age until they were slaughtered at 24 weeks of age.

### 2.7. Experimental Design for In Vivo Feeding Trial

For the present feeding trial, a juice mixture was prepared by mixing (*v*/*v*) equal volume of fermented rakkyo pickled juice and fermented rakkyo residual liquid. The juice mixture was added to the drinking water (*v*/*v*) at four different percentage to each of four designated study groups (Table 1). The first group (control or 0% mixture) of pigs were managed only with standard diet and drinking water but no pickled juice mixture. The second group (5% mixture) of pigs were fed with a 5% mixed pickled juice (2.5% fermented rakkyo pickled juice and 2.5% residual liquid) in drinking water. The third group (20% mixture) of pigs were fed with a 20% juice mixture (10% fermented rakkyo pickled juice and 10% residual liquid) in drinking water. The fourth group (40% mixture) of pigs were fed with a 40% juice mixture (20% fermented rakkyo pickled juice and 20% residual liquid) in drinking water. The feeding treatments were carried out from the time they were 4 weeks old to 17 weeks old.

### 2.8. Measuring Body Growth Rate

The body weight of all the study piglets was measured every week starting from the trial at 4 weeks of age until 24 weeks at slaughtering. Then average body weight gain was estimated to calculate the growth rate.

### 2.9. Blood Sampling and Cell Counting

The whole blood sample was collected in heparinized tube through venipuncture of all study pigs at 17 weeks of their age. Blood plasma were separated and stored at −20 °C until analyzing. Total number of white blood cell (WBC) was counted by using Celltac MEK-4100 (Nihon Kohden Co. Ltd., Tokyo, Japan) and the specific buffers. The granulocyte/lymphocyte ratio of peripheral blood was evaluated by estimating the percentage of each cell population as observed in a thin smear prepared from heparinized whole blood sample. Blood smear was prepared by using the d (Diff-Quik® reagents, Tokyo, Japan). Three repeated counting were carried out for each smear by using light microscope.

### 2.10. Western Blotting for Detecting Enteropathogenic Escherichia coli (ETEC)

In order to detect the presence of pathogenic *E. coli* in the fecal samples, western blotting was carried out using anti-ETEC K88 and anti-ETEC K99 fimbrial antisera (SSI 51172, SSI 51173, VERITAS Co., Tokyo, Japan), and anti-ETEC 987P fimbrial antisera (originally generated in rabbit immunized purified pili of ETEC987P) for the determination of each pilus. Horseradish peroxidase conjugated anti-rabbit IgG was used as secondary antibody (7074, Cell signaling Technology Japan, K.K., Tokyo, Japan). All procedures followed to a commercial kit, ECL western Blotting Detection System (GE Health care Co., Tokyo, Japan). The feces samples were stirred vigorously by sonication and separated by centrifugation for 5 min at 20 °C. the precipitation was dissolved by using Thermo Scientific Tissue Protein Extraction kit (Tokyo, Japan), and purified by centrifugation. The supernatant was subjected for membrane blotting.

### 2.11. Evaluation of Plasma C-Reactive Protein (CRP) Level

The plasma CRP concentration was measured by using the Fujifilm clinical chemical analyzer (Fujifilm Dri-Chem 3500i, Burladingen, Germany) following the manufacturer’s instruction.

### 2.12. Evaluation of Phagocytic Activity

The luminol reaction with oxygen radicals occurred from broken opsonized zymosan was detected and evaluated as phagocytic activity in the peripheral blood by using Fujifilm Luminescent Image Analyzer (LAS 3000, Fujifilm, Tokyo, Japan). The total luminol chemical reaction was measured sequentially and recognized as the area by the integration method. The measurements were repeated twice for every sample. The reaction was shown as relative light unit (RLU).

### 2.13. Evaluation of Complement Activity

Plasma alternative complement activity was evaluated as disruption degree of goat red blood cell (GRBC) by pig plasma complement protein. A volume of 150 μL of GRBC was added gently to a mixture of 30 μL of plasma and 270 μL of experimental buffer, and then the mixture was incubated at 37 °C for 40 min. After the inhibition of the reaction by using 4.05 mL of EDTA solution, the supernatant was obtained by centrifugation of the mixture at 4 °C for 10 min and separated quickly. The absorbance of the supernatant was determined at the web length of 542 nm and the optical density (OD) was recorded.

### 2.14. Evaluation of Carcass Weight and Quality

Immediately after slaughtering the study pigs at 24 weeks of their age, carcass weight and carcass quality estimates were recorded. The carcass quality was evaluated based on the standards those were set by the Japanese Meat Grading Association. The carcass meat was judged and ranked as high, middle, or mediocre classes and out of standards as described in our previous publication [18]. The weight was measured after dressing in the slaughterhouse of a market site, and carcass quality was evaluated there.

### 2.15. Statistical Analysis

The statistical analyses were performed by using SAS program (v. 9.1; Cary, NC, USA). The results are presented as means ± SDs of three independent measurements. Relative indices were estimated by comparing all means. The comparison for significant difference among means was carried out by the Tukey-Kramer method via ANOVA and regression model after fitting to a cubic expression. The *p* < 0.05 was considered as a significance threshold.

## 3. Results

### 3.1. Nutritional Composition of Fermented Rakkyo Pickled Juice and Residues

#### 3.1.1. Distribution of Major Nutrients

**Figure 1:**
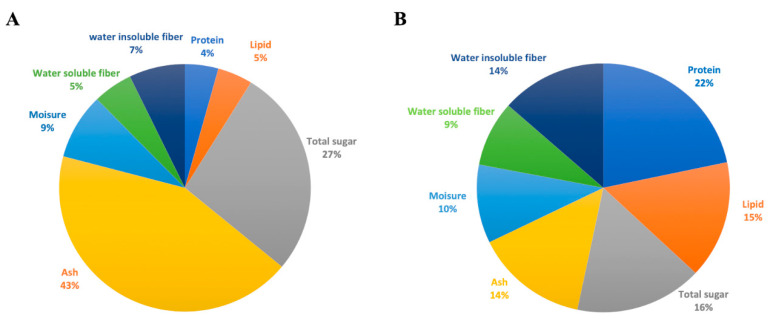
The nutritional composition of the fermented rakkyo. (**A**) The proportions of nutritional ingredients of the solution of fermented rakkyo residual powder. (**B**) The proportions of nutritional ingredients of fermented rakkyo residual liquid without adding dextrin sugar.

In order to analyze the effect of fermented rakkyo pickled residues on immunity and production performance of pigs, we first investigated the nutritional composition of fermented rakkyo pickled residual liquid and rakkyo residual powder. Each nutritional component of the solution of fermented rakkyo residual powder and fermented rakkyo residual liquid is presented as a percentage shown in the pie chart (Figure 1). A higher proportion of protein (22%) and lipid (15%) were estimated in the fermented rakkyo residual liquid as compared to the protein (4%) and lipid (5%) percentage of rakkyo residues (Figure 1A,B). On the other hand, the percentages of total sugar (16%) and ash (14%) in the fermented rakkyo residual liquid were much lower when compared to the proportion of sugar (27%) and ash (43%) in rakkyo residual powder (Figure 1A,B). The proportions of moisture, water soluble fiber, and water insoluble fiber were estimated as 9%, 5%, and 7%, respectively, in the fermented rakkyo residual powder, while they were measured as 10%, 9%, and 14%, respectively, in the fermented rakkyo residual liquid (Figure 1A,B).

#### 3.1.2. Individual Sugar Contents

The individual sugar content in a 100 g of sample taken from purified bacterial cells, polysaccharides derived from bacterial starter culture, fermented rakkyo pickled juice, and fermented rakkyo residual liquid were estimated as 4.9 g, 13.8 g, 1.3 g, and 5.5 g, respectively (Table 2). The fermented rakkyo pickled juice contained relatively less amount of total sugar as compared to fermented rakkyo residual liquid. The fermented rakkyo pickled juice contained only glucose and mannose, while the solution of fermented rakkyo residual liquid had arabinose, xylose, and galactose, in addition to glucose and mannose (Table 2).

The proportion of low molecular weight (<1000 g/mol) sugars was significantly higher (91%) in the fermented rakkyo pickled juice as compared to that of the fermented rakkyo residues solution (71%) (Table 2). The percentage of medium to high molecular weight (>1000 to <300,000 g/mol) sugar was significantly lower (7%) in fermented rakkyo pickled juice as compared to that of fermented rakkyo residues solution (26%). On the other hand, the proportion of very high molecular weight (>1,000,000 g/mol) sugars was also relatively lower (1%) in fermented rakkyo pickled juice as compared to that of the fermented rakkyo residual liquid (2%) (Table 2).

#### 3.1.3. Amino Acid Components

**Figure 2:**
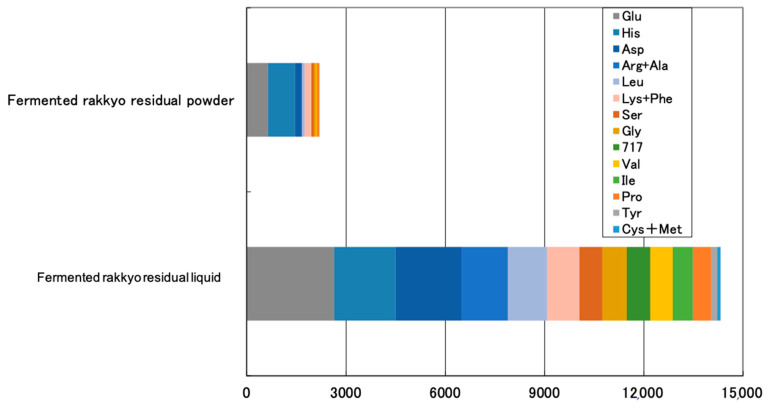
Amino acid components of fermented rakkyo residual liquid (pickled juice) and residual powder solution (mg/100 g).

The amino acid components of fermented pickled rakkyo residual liquid showed a remarkable difference when compared with that of the fermented residual powder (Figure 2). The fermented rakkyo residual powder sample showed the presence of eight amino acids: glutamine (658 mg/100 g), histidine (818 mg/100 g), aspartate (192 mg/100 g), leucine (82 mg/100 g), lysine + pheonine (202 mg/100 g), serine (86 mg/100 g), glycine (62 mg/100 g), valine (25 mg/100 g), and proline (74 mg/100 g). The levels of amino acids were significantly higher in the fermented rakkyo residual liquid as compared to that of fermented residual powder. The fermented rakkyo residual liquid showed 15 amino acids (including the eight contained in fermented residues): glutamine (2650 mg/100 g), histidine (1859 mg/100 g), aspartate (2005 mg/100 g), arginin + alanin (1383 mg/100 g), leucine (1188 mg/100 g), lysine + pheonine (974 mg/100 g), serine (700 mg/100 g), glycine (734 mg/100 g), threonine (717 mg/100 g), valine (664 mg/100 g), isoleucine (622 mg/100 g), proline (531 mg/100 g), tyrosine (201 mg/100 g), and cysteine + methionine (89 mg/100 g) (Figure 2).

### 3.2. Microbial Loads of Fermented Rakkyo Pickled Juice

The microbial growth was evaluated as colony forming unit (CFU) in the fermented rakkyo pickled juice. The general viable bacterial count in fermented rakkyo pickled juice was estimated as (1.1–4.5) × 10^8^. The heat-resistant bacteria growth was determined as 1.25 × 10^3^ to 1.9 × 10^5^, coliform counts was ranged from 5.0 × 10^3^ to 8.0 × 10^5^, fungal growth was estimated between 60 and 1400, and overall gram-negative bacterial growth was estimated as more than one million.

### 3.3. Effect of Immunobiotic Pickled Juice Feeding on Growth Rate

**Figure 3:**
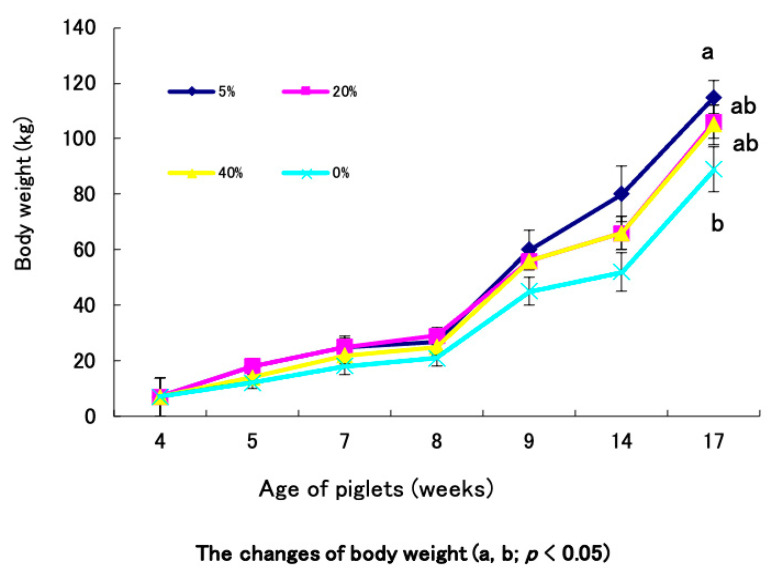
Body weight gain of study pigs over the feeding trial. The results presented are the mean ± SD of three independent experiments. Different letters among contrasting group indicate significant (a, b; *p* < 0.05) difference while same letter indicates no significance difference.

We first investigated the effect of immunobiotic pickled juice in body weight gain of the growing pigs. The pigs who ingested the 5% juice mixed with residue had the highest body weight among the four studied groups. The growth curve indicated that there was a significant difference of weight gain between 5% mixture of pickled juice and residue group compared to the control pigs (Figure 3). Both 5% mixture and 20% mixture groups showed an increasing growth trend than controls; however, differences between these two treatments were not statistically significant. The 20% mixture group and 40% mixture (20% rakkyo pickled juice and 20% rakkyo residue) group followed a similar trend of growth pattern over the studied period (Figure 3).

### 3.4. Effect of Immunobiotic Pickled Juice Feeding on Porcine Gut-Health

**Figure 4:**
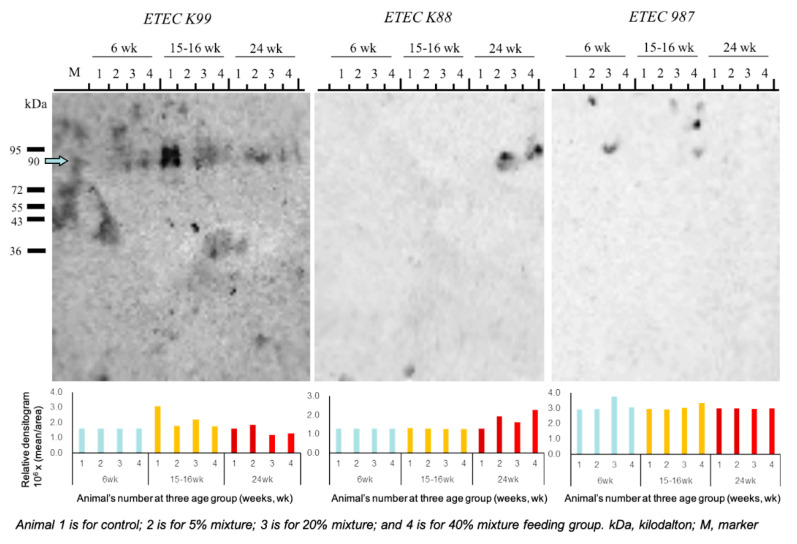
Western blot analysis for the detection of Enterotoxigenic *E. coli* (ETEC) strains in the feces of study pigs.

Enterotoxigenic *E. coli* is one of the major bacterial pathogens causing severe diarrhea in pigs. In order to evaluate the effect of immunobiotic feeding on gut-health status, the presence of diarrheic symptom and the western blot-based detection of K88, K99, and 987P ETEC strains in the fecal samples collected at 6, 16, and 24 weeks of age during the feeding trial were investigated. The pigs from the control group were affected with diarrhea at 16 weeks of their age, while none of three immunobiotic treated groups experienced any diarrhea (Figure 4). The western blot analysis confirmed that the pigs from the control group had enteric infections at 16 weeks of their age while the other three groups of immunobiotic treated pigs had no *E. coli* infection throughout the feeding trial period (Figure 4).

### 3.5. Effect of Immunobiotic Feeding on Immune Response Traits

#### 3.5.1. Plasma CRP Concentration

**Figure 5:**
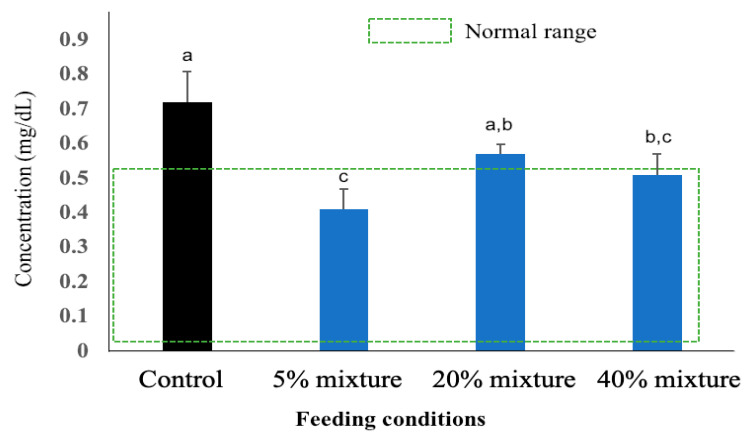
The plasma CRP level of pigs of all four study groups at 17 weeks of age. Results presented are the mean ± SD of three independent experiments. Different letters upon the bars indicate significant difference at 5% (*p* < 0.05) level while same letters indicate no significance difference when compared two bars.

The c-reactive protein (CRP) is a plasmatic protein of the pentraxin family and an acute phase reactant that is useful as a general inflammation marker. The plasma CRP levels in physiologically normal pigs ranges between 0.02 and 0.52 mg/dL [30]. The plasma CRP concentrations measured in pigs at 17 weeks of age are presented in Figure 5. The highest CRP levels were estimated in plasma of control pigs and the values were above the normal range. The pigs treated with 5% mixed pickled juice demonstrated the plasma CRP level within the normal ranges of estimates, and the value was significantly lower than control group and the group treated with 20% mixture of pickled juice with residue. The CRP level of pigs treated with 20% mixture of pickled juice and residue was estimated as slightly above the normal range, but significantly lower than the control group. The CRP level of pigs treated with 40% mixture (20% juice and 20% residue) pickled juice was estimated within the normal range but still significantly higher than the group treated with 5% mixture of pickled juice and residue. There was no significant difference in plasma CRP level between the group treated with 20% mixture and 40% mixture of residues and pickled juice (Figure 5).

#### 3.5.2. Phagocytosis and Complement Activity

**Figure 6:**
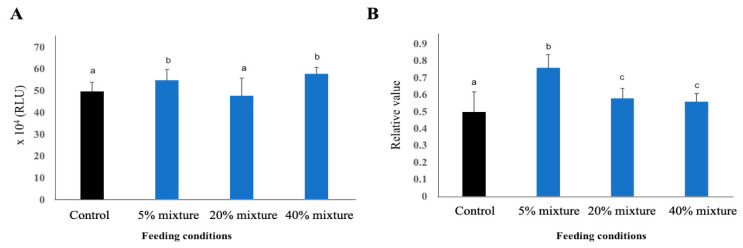
Phagocytic activity (**A**) and complement activity (**B**) in the blood of study pigs at 17 weeks of their age. Results presented are the mean ± SD of three independent experiments. Different letters upon the bars indicate significant difference at 5% (*p* < 0.05) while same letters indicate no significance difference when compared two bars.

A higher phagocytic activity of macrophages is an indicator of general inflammatory status of the body. The phagocytic activity of macrophage was increased in pigs treated with immunobiotic pickled juice compared to the pigs of control group (Figure 6A). Among the treated groups, the macrophage activity was higher in 5% mixture group and 40% mixture group than 20% mixture group (Figure 6A).

The activity of complement proteins in the blood cells increased in pigs treated with immunobiotic pickled juice compared to the pigs of control group (Figure 6B). Among the treated groups, the macrophage activity was higher in pigs treated with 5% mixture of pickled juice and residue followed by pigs treated with either 20% pickled juice mixed with residues, and those with 40% mixture (20% each of pickled juice and residue) group (Figure 6B).

#### 3.5.3. WBC Counts and the Ratio of Granulocyte and Lymphocytes

**Figure 7:**
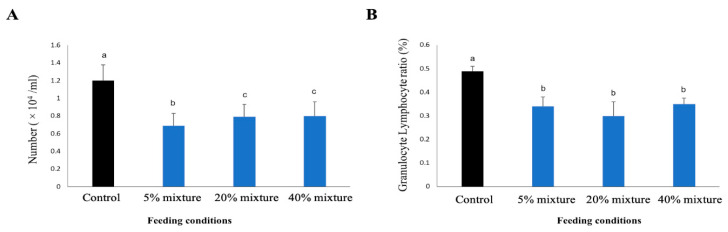
White bold Cell (WBC) count (**A**) and the ratio of granulocyte and lymphocyte (**B**) in the peripheral of study pigs at 17 weeks of their age. Results presented are the mean ± SD of three independent experiments. Different letters upon the bar indicate significant difference at 5% (*p* < 0.05) while same letters indicate no significance difference when compared two bars.

The increment of WBC count is an indicator of the presence of generalized infectious or noninfectious inflammatory stress in the body. The pigs tread with both 5% and 20% mixture of pickled juice with residues had WBC counts within the normal ranges of estimates, and the values were significantly lower than that of control group (Figure 7A). The WBC count showed an increasing trend in the group treated with 5% mixed pickled juice was the lowest among all study groups. The 40% mixture group showed WBC count lower than that of control group but higher than those of other treated groups, but none of the differences are statistically significant (Figure 7A).

A lower granulocyte and lymphocyte ratio of peripheral blood is associated with the presence of generalized either infectious or noninfectious inflammatory status of the body. All three treated groups of pigs showed significantly lower granulocyte/lymphocyte ration than the control group (Figure 7B). However, the variation of granulocyte/lymphocyte ratio among three treated groups was not significant.

### 3.6. Effect of Immunobiotic Feeding on Carcass Weight and Carcass Quality

**Figure 8:**
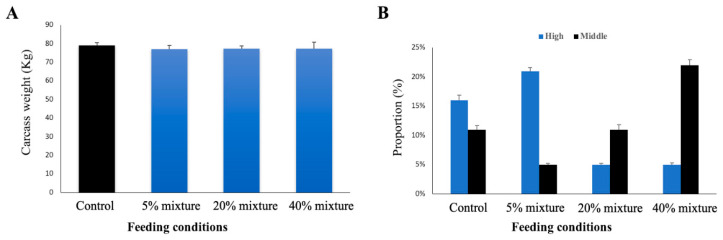
Carcass weight (**A**) and carcass quality (**B**) of different groups of study pigs at slaughtered at 24 weeks of age. Results presented are the mean ± SD of three independent experiments.

Carcass weight refers to body weight dressed after slaughter. It depends on the weight of a pig after it is partially butchered, all internal organs and the head are removed, along with the inedible (or less desirable) portions of the tail and legs. It includes the bones, cartilage, and other structures. At the of the feeding trial of 24 weeks, all study pigs were slaughtered, and the carcasses were weighted; the average carcass weight was estimated as 79.1, 77.0, 77.2, and 77.2 kg in 5% mixed, 20% mixed, 20% each, and control groups, respectively (Figure 8A). There was no significant difference in carcass yield among the study groups.

In order to determine the market price of pork, the overall carcass quality was ranked as high, medium or low, based on quality parameters measured at the slaughterhouse. Only the high-ranked and medium-ranked proportion of meat are shown in Figure 8B. In the control group, the proportion of high-ranked meat yield was higher than the proportion of medium-ranked meat. The proportion of high-ranked meat yield was significantly increased, and the medium-ranked proportion was significantly decreased in 5% mixture group when compared with control group (Figure 8B). On the other hand, both 20 mixture group and 40% mixture group, the ratio has significantly changed, the proportion of high-ranked meat yield was reduced, and the medium-ranked proportion was increased when compared with 5% mixture group as well as control group (Figure 8B).

## 4. Discussion

About one-third of the food produced annually worldwide ends up as waste. Food wastes are generated throughout the value chain from primary production to the final household consumptions. It has been estimated in the global food industry that around 1.3 billion tons of various food wastes are discarded every year [31]. In many cases, food wastes are difficult to reutilize for the recovery of value-added products for human consumption due to their biological instability, potential pathogenic hazards, high water content, rapid autoxidation, and high levels of enzymatic activity [32]. On the other hand, these biomaterials comprise rich nutrient stock [33] and could easily be utilized as animal feedstock. In order to explore sustainable management strategies for the wastes generated from industrial processing of rakkyo vegetable for fermented pickled dish, here, we investigated whether the spent pickled juice and the residual by-products can combinedly be utilized as feed supplement for promoting the health and production performance of growing pigs.

The nutritional composition of fermented rakkyo residual solution and the fermented pickled juice were estimated as 27% and 16%; 4% and 22%; 5% and 15%; 5% and 14%; 9% and 10%; of carbohydrate, protein, lipid, water-soluble fiber, and moisture, respectively. The discrepancies between the nutrition and protein contents of rakkyo reported in this study and values published elsewhere may be due to differences in variety and growing conditions [34]. The fermented rakkyo pickled juice contained high proportion of protein and lipid but lower proportion of carbohydrate as compared to that of fermented rakkyo residues. Steps of processing and preservation are known to affect the nutritional ingredients of pickled food stuff [4,11]. Monotano et al. [4] evaluated the effect of processing, with and without fermentation, on the nutritional composition of pickled garlic and reported that fermented pickled garlic had a higher content of riboflavin, α-tocopherol, and most individual amino acids but a lower thiamin level compared with that of fresh unfermented garlic. In a similar study, it was reported that contents of organosulfur compounds were also affected by the pickling process of garlic [11]. A high protein and low energy diet are associated with the health and immunocompetence of growing pigs [35]. One important fact to consider is that high protein becomes the first limiting factor in immune-challenged pigs because many components of the immune system are rich in proteins [35]. It is also generally accepted that energy requirements are lower for growing animals during a health challenge [36]. Therefore, a high protein diet would be beneficial for immunocompetent growing pigs. However, the effects of nutritional ingredients of spent pickled juice had a limited focused in health and production of pigs because of using only a small proportion as feed supplement, rather, its priority relied on being a suitable carrier for bioactive immunomodulatory components to be compatible with the intended feed formulation. To this end, the proportion of low molecular weight (<1000 g/mol) sugars was significantly higher (91%) in the fermented rakkyo pickled juice as compared to that of the unfermented rakkyo residual solution (71%). The sugars of low molecular weight can be dissolved in water at room temperature without difficulty; therefore, it would be convenient for practice in drinking water.

The LAB fermentation has been applied as a preservation method for vegetable products and is considered an important technology in food science [37], as these foods are well suited to promoting the positive health effects of probiotics [38]. Likewise, the application of LAB in the preservation and fermentation of livestock feedstock is also a well-known practice. For cattle production, an adequate and consistent supply of forage recourses is a priority, due to their high proportion in the ruminant’s daily feed formulation. To maintain an adequate supply of green grass in cold winter months or in adverse climatic conditions, ensiling is an efficient strategy for moist forage feedstuff preservation, based on natural lactic acid fermentation under anaerobic condition [39]. Several LAB strains (*L. buchneri, L. plantarum, and L. casei*) have been implicated for the preparation of fermented silage of whole corn crops that ensure the cost-effective feed formulation and promotes the animal’s production performance [39]. In particular, the *L. plantarum* N14 has been implicated in changes in immune parameters such as eosinophils and Th1 in humans [19,20,21]. Therefore, addition of LAB-enriched pickled juice in the feedstock would preserves the feed quality and promotes the animal health and performance when used as feed supplement.

Since *L. plantarum* can secrete EPS, the fermented rakkyo pickled juice not only carries immunobiotic *L. plantarum* N14 but also the secretory EPS. The EPS producing LAB strains including *L. plantarum* N14 are industrially important and have been used as starter cultures or conjugants to develop fermented foods such as yoghurt, cheese, pickles, and cereal-based products [40,41] due to their viscosity and mouth feel enhancement properties. EPS are widely used as natural bio-thickeners, especially due to their functional properties and potential immunomodulatory, immunostimulatory, antitumor, anti-inflammatory and antioxidant activities [42,43]. Accumulated reports have demonstrated that EPS produced by lactobacilli have immunoregulatory potentials [7,8]. Purified EPS produced by *L. rhamnosus* RW-9595M has reported to exert immunosuppressive properties in macrophages by inducing high level of IL-10 and low level of TNFα, IL-6 and IL-12 [42]. *L. plantarum* strains isolated from Chinese sauerkraut has reported to exert probiotic properties in vitro and cholesterol-lowering effect in vivo mice [7]. In addition, the EPS have been implicated as prebiotics by the intestinal bacteria to promote the proliferation of other probiotics [41]. Therefore, the *L. plantarum* N14 derived EPS content of the fermented pickled juice when used as feed supplement would contribute to enhance the mucosal immunity through promoting the growth and colonization of beneficial bacterial in the intestines of pigs.

In commercial swine production, sub-therapeutic doses of broad-spectrum antibiotics (e.g., chlortetracycline) have long been used as in-feed premix to promote growth and reduce the incidence of diarrhea [44,45]. However, in recent years, questions have arisen over the inclusion of in-feed antibiotics as they contribute to the antimicrobial resistance within the food animal production [46]. For instance, the addition of in-feed antibiotics to the diet of nursery pigs has been associated with an increased resistance of *E. coli* to antibiotics [47]. Moreover, due to legal restriction on the use of in-feed antibiotic growth promoters in European and developed countries, alternative technologies, such as direct feed probiotic supplements, are desired to reduce the use of in-feed antibiotics in nursery pigs’ diet. Immunobiotic microorganisms have been proposed as an alternative means to improve the health and protect against infections [48,49,50]. To this end, the utilization of spent pickled juice containing immunobiotics will not only provide a solution for food waste management, but also serve as value-added feed supplement for the healthy pig production without using in-feed antibiotics.

In this study, the untreated healthy control pigs had infected with gut pathogens *E. coli* infection and showed significantly higher plasma CRP level, and lower level of compliment and phagocytic activity in peripheral blood when compared to all groups pig fed with immunobiotic feed supplements. This may be due to the fact that immunobiotic *L. plantarum* N14 treated pigs received continuous low-grade immunomodulatory stimulations unlike the control pigs, who were given an antibiotic-free, basic diet. The untreated control pigs receiving no or minimum microbial exposure resulted in them having reduced inflammatory response mediators in the peripheral circulation, which in turn increased the complement and phagocytic activity as compared to the pigs provided with the immunobiotic treated feed. Immunobiotics are known to play a beneficial role in the prevention and therapy of a variety of intestinal inflammatory disorders [49,50]. It has been shown that pretreatment of piglets with *L. rhamnosus* ATCC7469 ameliorates F4^+^ETEC-induced diarrhea [51]. A significant elevated expression of jejunal TLR4 and IL-8 mRNA in piglets exposed to F4^+^ETEC; and the increased intestinal TLR4 and IL-8 mRNA expression was attenuated by pretreatment with the *L. rhamnosus* ATCC7469 strain [52]. Moreover, an induction of ileal NOD1 that was accompanied by upregulation of TLR2 and TLR9 expression in the pigs pretreated with the *L. rhamnosus*, suggesting that the anti-inflammatory effect of the ATCC7469 strain may be a result of synergistic response of TLR2, TLR9 and NOD1 [52]. In a previous in vivo study, we demonstrated that pretreatment with *L. jensenii* TL2937 led a reduced inflammatory marker in the blood [20], suggesting that the immunobiotic TL2937 strain would be able to avoid non-protective inflammation. The *L. jensenii* TL2937 significantly reduced blood complement activity and C-reactive protein concentrations while no changes were observed in blood leucocytes, ratio of granulocytes to lymphocyte numbers, phagocytic activity of macrophages [20]. Since *L. plantarum* N14 have already shown to exert the TLR-mediated anti-inflammatory effects on PIE cells [8], improving immune functions of pigs after the dietary intake of fermented pickled juice in the present study suggest that using *L. plantarum* N14-fermented bioactive pickled juice would be a useful functional feed supplement for swine ration.

The growth rate and carcass quality were enhanced by the immunobiotic-rich pickled juice when used as supplementation in the porcine feed formula. Though the average carcass yield was mostly similar, the pigs fed with 5% immunobiotic pickled juice produced significantly higher proportion of high-ranked pork as compared to that of untreated pigs. Recent studies have provided evidence for the influence of immunobiotic feeding on shear force, ash, salinity, and pH, as well as polyunsaturated fatty acids, ascorbic acid, some amino acids, and nucleotide compounds improvements related to the taste, flavor and quality of pork [14,16]. Dietary supplementation with probiotic Bacillus spp. prepared at 0.2% was found to be effective in improving the growth performance, carcass weight, and grade in pigs without affecting average daily feed intake [53]. It was reported that *L. plantarum* ZJ316 isolated from infant fecal sample are able to exert a probiotic effect on the porcine growth and pork quality [54]. It was also predicted that *L. plantarum* protect the host not by colonization and alteration of the gut bacterial community, but also by inhibiting the growth of opportunistic pathogens and promoting increased villus height [54]. Likewise, it has revealed in our previous in vivo study that *L. jensenii* TL2937 are able to significantly improve the growth performance, productivity, and carcass quality in post-weaning pigs when administered through feed supplement through reducing the TLR4-mediated inflammatory responses [18]. All these findings are in line with the results of present study, in particular the positive effects of *L. plantarum* N14 containing fermented pickled juice on growth rate and carcass quality of growing-finisher pigs.

## 5. Conclusions

The present in vivo immunobiotic feeding regime revealed that there are significant positive health benefits of rakkyo pickled juice fermented with *L. plantarum* N14 as feed supplements on the growth and production performance as well as immune health status of growing pigs. The results indicated that addition of both 5% and 20% mixture of fermented pickled juice and rakkyo residual liquid as dietary supplement have similar trends of beneficial effects in terms of complement, phagocytic activity, and leucocytes count in the peripheral blood, preventing enteropathogenic infection, and improving the growth rate and carcass quality of pigs. Therefore, the use of 5% mixture of rakkyo pickled juice fermented with *L. plantarum* N14 in the porcine feed formulation would be a cost-effective choice. This approach would contribute not only to the efficient management of food waste but also to utilize them as nutraceuticals for promoting animal health and production. However, further studies should focus on the dose optimization of *L. plantarum* N14 fermented pickled juice supplement in swine feed for economic considerations for mass scale application.

## Figures and Tables

**Table 1:** Study groups and dietary treatments used for the in vivo trial.

Study Groups	Dietary Treatment Given through Drinking Water
Control or 0% mixture	Only drinking water
5% mixture	Drinking water + 5% juice mixture (2.5% fermented rakkyo pickled juice and 2.5% residual liquid)
20% mixture	Drinking water + 20% juice mixture (10% fermented rakkyo pickled juice and 10% residual liquid)
40% mixture	Drinking water + 40% juice mixture (20% fermented rakkyo pickled juice and 20% residual liquid)

**Table 2:** Individual sugar composition and their category based on molecular weight (MW) in each 100 g sample.

Sugar Name	Pure Bacterial Cells	Polysaccharides of Bacterial Starter Culture	Fermented Rakkyo Pickled Juice	Fermented Rakkyo Residual Liquid
Glucose	2.1	5.6	0.9	2.8
Arabinose	-	-	-	0.3
Xylose	-	-	-	0.4
Mannose	-	4.4	0.4	-
Galactose	1.1	1.7	-	2
Rhamnose	0.5	0.4	-	-
Ribose	1.2	1.7	-	-
Fucose	-	-	-	-
Total (g)	4.9	13.8	1.3	5.5
**Range of MW**				
1,000,000 or more	2	2	1	2
300,000 ~	1	1	1	1
100,000 ~	-	5	1	-
30,000 ~	2	22	-	-
10,000 ~	2	19	-	2
3000 ~	3	15	1	8
1000 ~	21	15	5	16
<1000	69	21	91	71
Total (%)	100	100	100	100

## Data Availability

All the data related to this project are presented here.
